# Supplementary material for: Evaluation of Autof MS2600 and MBT Smart MALDI-TOF MS Systems for Routine Identification of Clinical Bacteria and Yeasts
Source: Microorganisms. 2024 Feb 13;12(2):382. doi: 10.3390/microorganisms12020382 (PMC10892063; doi:10.3390/microorganisms12020382)
Supplement: Supplementary file 1 [file microorganisms-12-00382-s001.zip › Supplementary table S1. Identification score for bacterial species.pdf]

| MS2600 ID | Score Autobio | MBT smart ID | Score Bruker | Sequence ID |
|---|---|---|---|---|
| *Achromobacter xylosoxidans* | 9.54 | *Achromobacter xylosoxidans* | 2.24 | |
| *Achromobacter xylosoxidans* | 9.56 | *Achromobacter xylosoxidans* | 2.07 | |
| *Acinetobacter baumannii* | 9.43 | *Acinetobacter baumannii* | 2.31 | |
| *Acinetobacter baumannii* | 9.69 | *Acinetobacter baumannii* | 2.33 | |
| *Acinetobacter baumannii* | 9.46 | *Acinetobacter baumannii* | 2.26 | |
| *Acinetobacter baumannii* | 9.66 | *Acinetobacter baumannii* | 2.29 | |
| *Acinetobacter baumannii* | 9.52 | *Acinetobacter baumannii* | 2.15 | |
| *Acinetobacter baumannii* | 9.64 | *Acinetobacter baumannii* | 2.36 | |
| *Acinetobacter baumannii* | 9.61 | *Acinetobacter baumannii* | 2.41 | |
| *Acinetobacter baumannii* | 9.67 | *Acinetobacter baumannii* | 2.41 | |
| *Acinetobacter baumannii* | 9.64 | *Acinetobacter baumannii* | 2.36 | |
| *Acinetobacter baumannii* | 9.34 | *Acinetobacter nosocomialis* | 2.23 | *Acinetobacter baumannii* |
| *Acinetobacter baumannii* | 9.61 | *Acinetobacter baumannii* | 2.31 | |
| *Actinomyces europaeus* | 9.14 | *Actinomyces europaeus* | 2.0 | |
| *Actinomyces neuii* | 9.45 | *Winkia neuii* | 2.29 | |
| *Actinomyces turicensis* | 9.09 | *Schaalia turicensis* | 2.09 | |
| *Actinomyces turicensis* | 9.3 | *Schaalia turicensis* | 2.06 | |
| *Aeromonas hydrophila* | 9.22 | *Aeromonas hydrophila* | 2.17 | |
| *Bacteroides faecis* | 9.55 | *Bacteroides faecis* | 2.3 | |
| *Bacteroides faecis* | 9.65 | *Bacteroides faecis* | 2.5 | |
| *Bacteroides fluxus* | 9.51 | *Bacteroides fluxus* | 2.38 | |
| *Bacteroidel fragilis* | 9.15 | *Bacteroides fragilis* | 2.15 | |
| *Bacteroides fragilis* | 9.7 | *Bacteroides fragilis* | 2.47 | |
| *Bacteroides fragilis* | 9.6 | *Bacteroides fragilis* | 2.42 | |
| *Bacteroides fragilis* | 9.7 | *Bacteroides fragilis* | 2.46 | |
| *Bacteroides fragilis* | 9.52 | *Bacteroides fragilis* | 2.29 | |
| *Bacteroides fragilis* | 9.72 | *Bacteroides fragilis* | 2.52 | |
| *Bacteroides fragilis* | 9.72 | *Bacteroides fragilis* | 2.5 | |
| *Bacteroides fragilis* | 9.71 | *Bacteroides fragilis* | 2.4 | |
| *Bacteroides ovatus* | 9.46 | *Bacteroides ovatus* | 2.14 | |
| *Bacteroides ovatus* | 9.62 | *Bacteroides ovatus* | 2.05 | |
| *Bacteroides thetaiotaomicron* | 9.52 | *Bacteroides thetaiotaomicron* | 2.26 | |

| | | | | |
|---|---|---|---|---|
| *Bacteroides thetaiotaomicron* | 9.56 | *Bacteroides thetaiotaomicron* | 2.12 | |
| *Bifidobacterium bifidum* | 9.16 | *Bifidobacterium bifidum* | 2.0 | |
| *Burkholderia gladioli* | 9.57 | *Burkholderia gladioli* | 2.36 | |
| *Citrobacter freundii* | 9.16 | *Citrobacter freundii* | 2.38 | |
| *Citrobacter freundii* | 9.0 | *Citrobacter braakii* | 2.23 | *Citrobacter freundii* |
| *Citrobacter koseri* | 9.41 | *Citrobacter koseri* | 2.44 | |
| *Clostridium hathewayi* | 9.53 | *Hungatella hathewayi* | 2.22 | |
| *Clostridium perfringens* | 9.71 | *Clostridium perfringens* | 2.56 | |
| *Clostridium ramosum* | 9.56 | *Clostridium ramosum* | 2.4 | |
| *Clostridium septicum* | 9.52 | *Clostridium septicum* | 2.16 | |
| *Corynebacterium striatum* | 9.26 | *Corynebacterium striatum* | 2.12 | |
| *Corynebacterium striatum* | 9.65 | *Corynebacterium striatum* | 2.2 | |
| *Corynebacterium striatum* | 9.63 | *Corynebacterium striatum* | 2.38 | |
| *Corynebacterium striatum* | 9.71 | *Corynebacterium striatum* | 2.31 | |
| *Corynebacterium striatum* | 9.74 | *Corynebacterium striatum* | 2.31 | |
| *Cutibacterium acnes subsp. acnes* | 9.16 | *Cutibacterium acnes* | 2.39 | |
| *Cutibacterium acnes subsp. acnes* | 9.06 | *Cutibacterium acnes* | 2.17 | |
| *Dermabacter hominis* | 9.14 | *Dermabacter hominis* | 2.08 | |
| *Dermabacter hominis* | 9.19 | *Dermabacter hominis* | 2.13 | |
| *Dermabacter hominis* | 9.13 | *Dermabacter hominis* | 2.15 | |
| *Eggerthella lenta* | 9.16 | *Eggerthella lenta* | 2.18 | |
| *Enterobacter cloacae* | 9.16 | *Enterobacter cloacae* | 2.16 | |
| *Enterobacter cloacae* | 9.04 | *Enterobacter cloacae* | 2.31 | |
| *Enterobacter cloacae* | 9.21 | *Enterobacter hormaechei* | 2.44 | *Enterobacter hormaechei* |
| *Enterobacter cloacae* | 9.01 | *Enterobacter cloacae* | 2.5 | |
| *Enterobacter cloacae* | 9.03 | *Enterobacter cloacae* | 2.39 | |
| *Enterobacter hormaechei* | 9.01 | *Enterobacter hormaechei* | 2.45 | |
| *Enterobacter hormaechei* | 9.08 | *Enterobacter hormaechei* | 2.28 | |
| *Enterobacter hormaechei* | 9.15 | *Enterobacter hormaechei* | 2.24 | |
| *Enterobacter kobei* | 9.17 | *Enterobacter kobei* | 2.39 | |
| *Enterobacter kobei* | 9.29 | *Enterobacter kobei* | 2.26 | |
| *Enterobacter kobei* | 9.09 | *Enterobacter kobei* | 2.29 | |
| *Enterobacter kobei* | 9.03 | *Enterobacter kobei* | 2.33 | |

| | | | |
|---|---|---|---|
| *Enterococcus avium* | 9.55 | *Enterococcus avium* | 2.01 |
| *Enterococcus avium* | 9.57 | *Enterococcus avium* | 2.19 |
| *Enterococcus faecalis* | 9.52 | *Enterococcus faecalis* | 2.19 |
| *Enterococcus faecalis* | 9.56 | *Enterococcus faecalis* | 2.2 |
| *Enterococcus faecalis* | 9.64 | *Enterococcus faecalis* | 2.21 |
| *Enterococcus faecalis* | 9.68 | *Enterococcus faecalis* | 2.40 |
| *Enterococcus faecalis* | 9.56 | *Enterococcus faecalis* | 2.08 |
| *Enterococcus faecalis* | 9.57 | *Enterococcus faecalis* | 2.21 |
| *Enterococcus faecalis* | 9.52 | *Enterococcus faecalis* | 2.15 |
| *Enterococcus faecalis* | 9.63 | *Enterococcus faecalis* | 2.25 |
| *Enterococcus faecalis* | 9.67 | *Enterococcus faecalis* | 2.39 |
| *Enterococcus faecalis* | 9.63 | *Enterococcus faecalis* | 2.29 |
| *Enterococcus faecalis* | 9.52 | *Enterococcus faecalis* | 2.07 |
| *Enterococcus faecalis* | 9.69 | *Enterococcus faecalis* | 2.37 |
| *Enterococcus faecalis* | 9.65 | *Enterococcus faecalis* | 2.36 |
| *Enterococcus faecalis* | 9.62 | *Enterococcus faecalis* | 2.18 |
| *Enterococcus faecalis* | 9.55 | *Enterococcus faecalis* | 2.17 |
| *Enterococcus faecalis* | 9.71 | *Enterococcus faecalis* | 2.24 |
| *Enterococcus faecalis* | 9.69 | *Enterococcus faecalis* | 2.28 |
| *Enterococcus faecalis* | 9.73 | *Enterococcus faecalis* | 2.46 |
| *Enterococcus faecalis* | 9.73 | *Enterococcus faecalis* | 2.36 |
| *Enterococcus faecalis* | 9.24 | *Enterococcus faecalis* | 2.3 |
| *Enterococcus faecalis* | 9.61 | *Enterococcus faecalis* | 2.05 |
| *Enterococcus faecalis* | 9.69 | *Enterococcus faecalis* | 2.25 |
| *Enterococcus faecalis* | 9.66 | *Enterococcus faecalis* | 2.21 |
| *Enterococcus faecalis* | 9.62 | *Enterococcus faecalis* | 2.16 |
| *Enterococcus faecalis* | 9.63 | *Enterococcus faecalis* | 2.16 |
| *Enterococcus faecalis* | 9.40 | *Enterococcus faecalis* | 2.01 |
| *Enterococcus faecalis* | 9.62 | *Enterococcus faecalis* | 2.18 |
| *Enterococcus faecalis* | 9.66 | *Enterococcus faecalis* | 2.08 |
| *Enterococcus faecalis* | 9.67 | *Enterococcus faecalis* | 2.3 |
| *Enterococcus faecalis* | 9.68 | *Enterococcus faecalis* | 2.28 |
| *Enterococcus faecalis* | 9.71 | *Enterococcus faecalis* | 2.21 |

| | | | |
|---|---|---|---|
| *Enterococcus faecium* | 9.67 | *Enterococcus faecium* | 2.39 |
| *Enterococcus faecium* | 9.63 | *Enterococcus faecium* | 2.32 |
| *Enterococcus faecium* | 9.56 | *Enterococcus faecium* | 2.05 |
| *Enterococcus faecium* | 9.64 | *Enterococcus faecium* | 2.32 |
| *Enterococcus faecium* | 9.67 | *Enterococcus faecium* | 2.19 |
| *Enterococcus faecium* | 9.43 | *Enterococcus faecium* | 2.16 |
| *Enterococcus faecium* | 9.6 | *Enterococcus faecium* | 2.42 |
| *Enterococcus faecium* | 9.64 | *Enterococcus faecium* | 2.37 |
| *Enterococcus faecium* | 9.6 | *Enterococcus faecium* | 2.43 |
| *Enterococcus faecium* | 9.3 | *Enterococcus faecium* | 2.05 |
| *Enterococcus faecium* | 9.6 | *Enterococcus faecium* | 2.29 |
| *Enterococcus faecium* | 9.52 | *Enterococcus faecium* | 2.24 |
| *Enterococcus faecium* | 9.59 | *Enterococcus faecium* | 2.3 |
| *Enterococcus faecium* | 9.67 | *Enterococcus faecium* | 2.47 |
| *Enterococcus faecium* | 9.66 | *Enterococcus faecium* | 2.48 |
| *Enterococcus faecium* | 9.56 | *Enterococcus faecium* | 2.41 |
| *Enterococcus faecium* | 9.67 | *Enterococcus faecium* | 2.35 |
| *Enterococcus faecium* | 9.67 | *Enterococcus faecium* | 2.41 |
| *Enterococcus faecium* | 9.62 | *Enterococcus faecium* | 2.42 |
| *Enterococcus faecium* | 9.67 | *Enterococcus faecium* | 2.3 |
| *Enterococcus faecium* | 9.49 | *Enterococcus faecium* | 2.19 |
| *Enterococcus faecium* | 9.54 | *Enterococcus faecium* | 2.35 |
| *Enterococcus faecium* | 9.77 | *Enterococcus faecium* | 2.49 |
| *Enterococcus faecium* | 9.67 | *Enterococcus faecium* | 2.48 |
| *Enterococcus faecium* | 9.59 | *Enterococcus faecium* | 2.28 |
| *Enterococcus gallinarum* | 9.67 | *Enterococcus gallinarum* | 2.37 |
| *Enterococcus gallinarum* | 9.57 | *Enterococcus gallinarum* | 2.35 |
| *Enterococcus gallinarum* | 9.35 | *Enterococcus gallinarum* | 2.03 |
| *Enterococcus malodoratus* | 9.6 | *Enterococcus malodoratus* | 2.14 |
| *Escherichia coli* | 9.62 | *Escherichia coli* | 2.28 |
| *Escherichia coli* | 9.73 | *Escherichia coli* | 2.34 |
| *Escherichia coli* | 9.62 | *Escherichia coli* | 2.31 |
| *Escherichia coli* | 9.56 | *Escherichia coli* | 2.21 |

| | | | |
|---|---|---|---|
| *Escherichia coli* | 9.58 | *Escherichia coli* | 2.14 |
| *Escherichia coli* | 9.7 | *Escherichia coli* | 2.35 |
| *Escherichia coli* | 9.54 | *Escherichia coli* | 2.28 |
| *Escherichia coli* | 9.67 | *Escherichia coli* | 2.28 |
| *Escherichia coli* | 9.55 | *Escherichia coli* | 2.06 |
| *Escherichia coli* | 9.5 | *Escherichia coli* | 2.38 |
| *Escherichia coli* | 9.13 | *Escherichia coli* | 2.2 |
| *Escherichia coli* | 9.60 | *Escherichia coli* | 2.21 |
| *Escherichia coli* | 9.32 | *Escherichia coli* | 2.32 |
| *Escherichia coli* | 9.42 | *Escherichia coli* | 2.09 |
| *Escherichia coli* | 9.45 | *Escherichia coli* | 2.09 |
| *Escherichia coli* | 9.63 | *Escherichia coli* | 2.19 |
| *Escherichia coli* | 9.59 | *Escherichia coli* | 2.3 |
| *Escherichia coli* | 9.65 | *Escherichia coli* | 2.16 |
| *Escherichia coli* | 9.62 | *Escherichia coli* | 2.18 |
| *Escherichia coli* | 9.49 | *Escherichia coli* | 2.27 |
| *Escherichia coli* | 9.57 | *Escherichia coli* | 2.09 |
| *Escherichia coli* | 9.6 | *Escherichia coli* | 2.41 |
| *Escherichia coli* | 9.7 | *Escherichia coli* | 2.34 |
| *Escherichia coli* | 9.75 | *Escherichia coli* | 2.4 |
| *Escherichia coli* | 9.34 | *Escherichia coli* | 2.3 |
| *Escherichia coli* | 9.59 | *Escherichia coli* | 2.19 |
| *Escherichia coli* | 9.63 | *Escherichia coli* | 2.04 |
| *Escherichia coli* | 9.72 | *Escherichia coli* | 2.4 |
| *Escherichia coli* | 9.68 | *Escherichia coli* | 2.4 |
| *Escherichia coli* | 9.66 | *Escherichia coli* | 2.2 |
| *Escherichia coli* | 9.6 | *Escherichia coli* | 2.36 |
| *Escherichia coli* | 9.65 | *Escherichia coli* | 2.05 |
| *Escherichia coli* | 9.68 | *Escherichia coli* | 2.09 |
| *Escherichia coli* | 9.48 | *Escherichia coli* | 1.83 |
| *Escherichia coli* | 9.68 | *Escherichia coli* | 2.32 |
| *Escherichia coli* | 9.6 | *Escherichia coli* | 2.05 |
| *Escherichia coli* | 9.44 | *Escherichia coli* | 2.0 |

| | | | |
|---|---|---|---|
| *Escherichia coli* | 9.67 | *Escherichia coli* | 2.26 |
| *Escherichia coli* | 9.66 | *Escherichia coli* | 2.11 |
| *Escherichia coli* | 9.52 | *Escherichia coli* | 2.07 |
| *Escherichia coli* | 9.5 | *Escherichia coli* | 2.04 |
| *Haemophilus haemolyticus* | 9.43 | *Haemophilus haemolyticus* | 2.18 |
| *Haemophilus haemolyticus* | 9.29 | *Haemophilus haemolyticus* | 2.19 |
| *Haemophilus influenzae* | 9.42 | *Haemophilus influenzae* | 2.24 |
| *Haemophilus influenzae* | 9.04 | *Haemophilus influenzae* | 2.12 |
| *Haemophilus influenzae* | 9.00 | *Haemophilus influenzae* | 2.1 |
| *Haemophilus influenzae* | 9.28 | *Haemophilus influenzae* | 2.2 |
| *Hafnia alvei* | 9.57 | *Hafnia alvei* | 2.27 |
| *Hafnia alvei* | 9.26 | *Hafnia alvei* | 2.11 |
| *Klebsiella aerogenes* | 9.55 | *Klebsiella aerogenes* | 2.19 |
| *Klebsiella aerogenes* | 9.56 | *Klebsiella aerogenes* | 2.15 |
| *Klebsiella oxytoca* | 9.43 | *Klebsiella oxytoca* | 2.1 |
| *Klebsiella oxytoca* | 9.48 | *Klebsiella oxytoca* | 2.16 |
| *Klebsiella oxytoca* | 9.64 | *Klebsiella oxytoca* | 2.33 |
| *Klebsiella oxytoca* | 9.09 | *Klebsiella oxytoca* | 2.28 |
| *Klebsiella oxytoca* | 9.5 | *Klebsiella oxytoca* | 2.31 |
| *Klebsiella oxytoca* | 9.61 | *Klebsiella oxytoca* | 2.17 |
| *Klebsiella pneumoniae* | 9.03 | *Klebsiella pneumoniae* | 2.27 |
| *Klebsiella pneumoniae* | 9.12 | *Klebsiella pneumoniae* | 2.34 |
| *Klebsiella pneumoniae* | 9.25 | *Klebsiella pneumoniae* | 2.34 |
| *Klebsiella pneumoniae* | 9.53 | *Klebsiella pneumoniae* | 2.24 |
| *Klebsiella pneumoniae* | 9.73 | *Klebsiella pneumoniae* | 2.54 |
| *Klebsiella pneumoniae* | 9.51 | *Klebsiella pneumoniae* | 2.43 |
| *Klebsiella pneumoniae* | 9.03 | *Klebsiella pneumoniae* | 2.3 |
| *Klebsiella pneumoniae* | 9.23 | *Klebsiella pneumoniae* | 2.07 |
| *Klebsiella pneumoniae* | 9.11 | *Klebsiella pneumoniae* | 2.45 |
| *Klebsiella pneumoniae* | 9.05 | *Klebsiella pneumoniae* | 2.31 |
| *Klebsiella pneumoniae* | 9.16 | *Klebsiella pneumoniae* | 2.4 |
| *Klebsiella pneumoniae* | 9.05 | *Klebsiella pneumoniae* | 2.41 |
| *Klebsiella pneumoniae* | 9.13 | *Klebsiella pneumoniae* | 2.37 |

| | | | | |
|---|---|---|---|---|
| *Klebsiella pneumoniae* | 9.22 | *Klebsiella pneumoniae* | 2.21 | |
| *Klebsiella pneumoniae* | 9.2 | *Klebsiella pneumoniae* | 2.29 | |
| *Klebsiella pneumoniae* | 9.01 | *Klebsiella pneumoniae* | 2.43 | |
| *Klebsiella pneumoniae* | 9.16 | *Klebsiella pneumoniae* | 2.07 | |
| *Klebsiella pneumoniae* | 9.12 | *Klebsiella pneumoniae* | 2.48 | |
| *Klebsiella pneumoniae* | 9.02 | *Klebsiella pneumoniae* | 2.34 | |
| *Klebsiella pneumoniae* | 9.03 | *Klebsiella pneumoniae* | 2.41 | |
| *Klebsiella pneumoniae* | 9.2 | *Klebsiella variicola* | 2.11 | *Klebsiella pneumoniae* |
| *Klebsiella pneumoniae* | 9.18 | *Klebsiella pneumoniae* | 2.01 | |
| *Klebsiella pneumoniae* | 9.17 | *Klebsiella pneumoniae* | 2.25 | |
| *Klebsiella pneumoniae* | 9.32 | *Klebsiella pneumoniae* | 2.34 | |
| *Klebsiella pneumoniae* | 9.68 | *Klebsiella pneumoniae* | 2.28 | |
| *Klebsiella pneumoniae* | 9.17 | *Klebsiella pneumoniae* | 2.33 | |
| *Klebsiella pneumoniae* | 9.72 | *Klebsiella pneumoniae* | 2.41 | |
| *Klebsiella pneumoniae* | 9.67 | *Klebsiella pneumoniae* | 2.51 | |
| *Klebsiella pneumoniae* | 9.20 | *Klebsiella pneumoniae* | 2.16 | |
| *Klebsiella pneumoniae* | 9.15 | *Klebsiella pneumoniae* | 2.45 | |
| *Klebsiella pneumoniae* | 9.28 | *Klebsiella pneumoniae* | 2.48 | |
| *Klebsiella pneumoniae subsp. pneumoniae* | 9.06 | *Klebsiella pneumoniae* | 2.49 | |
| *Klebsiella pneumoniae subsp. pneumoniae* | 9.13 | *Klebsiella pneumoniae* | 2.37 | |
| *Klebsiella pneumoniae subsp.ozaenae* | 9.02 | *Klebsiella pneumoniae* | 2.2 | |
| *Klebsiella pneumoniae subsp.ozaenae* | 9.02 | *Klebsiella pneumoniae* | 2.14 | |
| *Klebsiella variicola* | 9.07 | *Klebsiella variicola* | 2.32 | |
| *Lacticaseibacillus rhamnosus* | 9.62 | *Lacticaseibacillus rhamnosus* | 2.34 | |
| *Lactobacillus delbrueckii* | 9.01 | *Lactobacillus delbrueckii* | 2.25 | |
| *Lactobacillus delbrueckii* | 9.05 | *Lactobacillus delbrueckii* | 2.08 | |
| *Lactobacillus gasseri* | 9.53 | *Lactobacillus gasseri* | 2.25 | |
| *Lactobacillus rhamnosus* | 9.19 | *Lactobacillus rhamnosus* | 2.08 | |
| *Lactobacillus rhamnosus* | 9.6 | *Lactobacillus rhamnosus* | 2.35 | |
| *Lactobacillus rhamnosus* | 9.5 | *Lactobacillus rhamnosus* | 2.15 | |
| *Lactobacillus rhamnosus* | 9.49 | *Lacticaseibacillus rhamnosus* | 2.22 | |
| *Morganella morganii* | 9.64 | *Morganella morganii* | 2.12 | |
| *Morganella morganii* | 9.63 | *Morganella morganii* | 2.27 | |

| | | | |
|---|---|---|---|
| *Morganella morganii* | 9.6 | *Morganella morganii* | 2.1 |
| *Morganella morganii* | 9.73 | *Morganella morganii* | 2.43 |
| *Morganella morganii* | 9.75 | *Morganella morganii* | 2.43 |
| *Morganella morganii* | 9.77 | *Morganella morganii* | 2.57 |
| *Morganella morganii* | 9.69 | *Morganella morganii* | 2.39 |
| *Morganella morganii* | 9.75 | *Morganella morganii* | 2.41 |
| *Morganella morganii* | 9.75 | *Morganella morganii* | 2.55 |
| *Pantoea ananatis* | 9.51 | *Pantoea ananatis* | 2.15 |
| *Parvimonas micra* | 9.11 | *Parvimonas micra* | 2.3 |
| *Peptostreptococcus anaerobius* | 9.53 | *Peptostreptococcus anaerobius* | 2.19 |
| *Prevotella bivia* | 9.58 | *Prevotella bivia* | 2.09 |
| *Prevotella bivia* | 9.52 | *Prevotella bivia* | 2.14 |
| *Proteus mirabilis* | 9.68 | *Proteus mirabilis* | 2.42 |
| *Proteus mirabilis* | 9.65 | *Proteus mirabilis* | 2.38 |
| *Proteus mirabilis* | 9.75 | *Proteus mirabilis* | 2.39 |
| *Proteus mirabilis* | 9.63 | *Proteus mirabilis* | 2.22 |
| *Proteus mirabilis* | 9.63 | *Proteus mirabilis* | 2.4 |
| *Proteus mirabilis* | 9.24 | *Proteus mirabilis* | 2.5 |
| *Proteus mirabilis* | 9.6 | *Proteus mirabilis* | 2.14 |
| *Proteus mirabilis* | 9.67 | *Proteus mirabilis* | 2.24 |
| *Proteus mirabilis* | 9.65 | *Proteus mirabilis* | 2.4 |
| *Proteus mirabilis* | 9.7 | *Proteus mirabilis* | 2.35 |
| *Proteus mirabilis* | 9.71 | *Proteus mirabilis* | 2.36 |
| *Proteus mirabilis* | 9.02 | *Proteus mirabilis* | 2.46 |
| *Proteus mirabilis* | 9.67 | *Proteus mirabilis* | 2.46 |
| *Proteus mirabilis* | 9.66 | *Proteus mirabilis* | 2.29 |
| *Proteus mirabilis* | 9.55 | *Proteus mirabilis* | 2.19 |
| *Proteus mirabilis* | 9.63 | *Proteus mirabilis* | 2.29 |
| *Proteus mirabilis* | 9.73 | *Proteus mirabilis* | 2.42 |
| *Proteus mirabilis* | 9.69 | *Proteus mirabilis* | 2.44 |
| *Proteus mirabilis* | 9.73 | *Proteus mirabilis* | 2.54 |
| *Proteus mirabilis* | 9.72 | *Proteus mirabilis* | 2.46 |
| *Proteus mirabilis* | 9.62 | *Proteus mirabilis* | 2.31 |

| | | | |
|---|---|---|---|
| *Proteus mirabilis* | 9.7 | *Proteus mirabilis* | 2.51 |
| *Proteus mirabilis* | 9.67 | *Proteus mirabilis* | 2.29 |
| *Proteus mirabilis* | 9.69 | *Proteus mirabilis* | 2.39 |
| *Proteus mirabilis* | 9.69 | *Proteus mirabilis* | 2.39 |
| *Providencia rettgeri* | 9.45 | *Providencia rettgeri* | 2.05 |
| *Providencia stuartii* | 9.57 | *Providencia stuartii* | 2.43 |
| *Providencia stuartii* | 9.60 | *Providencia stuartii* | 2.35 |
| *Providencia stuartii* | 9.65 | *Providencia stuartii* | 2.47 |
| *Pseudomonas aeruginosa* | 9.68 | *Pseudomonas aeruginosa* | 2.34 |
| *Pseudomonas aeruginosa* | 9.66 | *Pseudomonas aeruginosa* | 2.37 |
| *Pseudomonas aeruginosa* | 9.63 | *Pseudomonas aeruginosa* | 2.33 |
| *Pseudomonas aeruginosa* | 9.51 | *Pseudomonas aeruginosa* | 2.23 |
| *Pseudomonas aeruginosa* | 9.6 | *Pseudomonas aeruginosa* | 2.33 |
| *Pseudomonas aeruginosa* | 9.68 | *Pseudomonas aeruginosa* | 2.19 |
| *Pseudomonas aeruginosa* | 9.66 | *Pseudomonas aeruginosa* | 2.36 |
| *Pseudomonas aeruginosa* | 9.65 | *Pseudomonas aeruginosa* | 2.03 |
| *Pseudomonas aeruginosa* | 9.41 | *Pseudomonas aeruginosa* | 2.2 |
| *Pseudomonas aeruginosa* | 9.51 | *Pseudomonas aeruginosa* | 2.21 |
| *Pseudomonas aeruginosa* | 9.55 | *Pseudomonas aeruginosa* | 2.31 |
| *Pseudomonas aeruginosa* | 9.67 | *Pseudomonas aeruginosa* | 2.27 |
| *Pseudomonas aeruginosa* | 9.52 | *Pseudomonas aeruginosa* | 2.22 |
| *Pseudomonas aeruginosa* | 9.57 | *Pseudomonas aeruginosa* | 2.31 |
| *Pseudomonas aeruginosa* | 9.52 | *Pseudomonas aeruginosa* | 2.14 |
| *Pseudomonas aeruginosa* | 9.6 | *Pseudomonas aeruginosa* | 2.37 |
| *Pseudomonas aeruginosa* | 9.68 | *Pseudomonas aeruginosa* | 2.49 |
| *Pseudomonas aeruginosa* | 9.5 | *Pseudomonas aeruginosa* | 2.29 |
| *Pseudomonas aeruginosa* | 9.64 | *Pseudomonas aeruginosa* | 2.26 |
| *Pseudomonas aeruginosa* | 9.58 | *Pseudomonas aeruginosa* | 2.3 |
| *Pseudomonas aeruginosa* | 9.46 | *Pseudomonas aeruginosa* | 2.16 |
| *Pseudomonas aeruginosa* | 9.56 | *Pseudomonas aeruginosa* | 2.27 |
| *Pseudomonas aeruginosa* | 9.53 | *Pseudomonas aeruginosa* | 2.1 |
| *Pseudomonas aeruginosa* | 9.7 | *Pseudomonas aeruginosa* | 2.35 |
| *Pseudomonas aeruginosa* | 9.65 | *Pseudomonas aeruginosa* | 2.43 |

| | | | |
|---|---|---|---|
| *Pseudomonas aeruginosa* | 9.64 | *Pseudomonas aeruginosa* | 2.31 |
| *Pseudomonas aeruginosa* | 9.54 | *Pseudomonas aeruginosa* | 2.09 |
| *Pseudomonas aeruginosa* | 9.67 | *Pseudomonas aeruginosa* | 2.32 |
| *Pseudomonas aeruginosa* | 9.60 | *Pseudomonas aeruginosa* | 2.35 |
| *Pseudomonas aeruginosa* | 9.66 | *Pseudomonas aeruginosa* | 2.36 |
| *Serratia marcescens* | 9.68 | *Serratia marcescens* | 2.26 |
| *Serratia marcescens* | 9.54 | *Serratia marcescens* | 2.13 |
| *Staphylococcus aureus* | 9.6 | *Staphylococcus aureus* | 2.27 |
| *Staphylococcus aureus* | 9.53 | *Staphylococcus aureus* | 2.33 |
| *Staphylococcus aureus* | 9.62 | *Staphylococcus aureus* | 2.43 |
| *Staphylococcus aureus* | 9.61 | *Staphylococcus aureus* | 2.34 |
| *Staphylococcus aureus* | 9.6 | *Staphylococcus aureus* | 2.11 |
| *Staphylococcus aureus* | 9.14 | *Staphylococcus aureus* | 2.09 |
| *Staphylococcus aureus* | 9.56 | *Staphylococcus aureus* | 2.23 |
| *Staphylococcus aureus* | 9.29 | *Staphylococcus aureus* | 2.04 |
| *Staphylococcus aureus* | 9.01 | *Staphylococcus aureus* | 2.03 |
| *Staphylococcus aureus* | 9.52 | *Staphylococcus aureus* | 2.12 |
| *Staphylococcus aureus* | 9.63 | *Staphylococcus aureus* | 2.38 |
| *Staphylococcus aureus* | 9.57 | *Staphylococcus aureus* | 2.23 |
| *Staphylococcus aureus* | 9.47 | *Staphylococcus aureus* | 2.34 |
| *Staphylococcus aureus* | 9.08 | *Staphylococcus aureus* | 2.04 |
| *Staphylococcus aureus* | 9.52 | *Staphylococcus aureus* | 2.23 |
| *Staphylococcus aureus* | 9.01 | *Staphylococcus aureus* | 2.15 |
| *Staphylococcus aureus* | 9.55 | *Staphylococcus aureus* | 2.32 |
| *Staphylococcus aureus* | 9.06 | *Staphylococcus aureus* | 2.09 |
| *Staphylococcus aureus* | 9.42 | *Staphylococcus aureus* | 2.24 |
| *Staphylococcus aureus* | 9.63 | *Staphylococcus aureus* | 2.4 |
| *Staphylococcus aureus* | 9.55 | *Staphylococcus aureus* | 2.34 |
| *Staphylococcus aureus* | 9.66 | *Staphylococcus aureus* | 2.41 |
| *Staphylococcus aureus* | 9.41 | *Staphylococcus aureus* | 2.3 |
| *Staphylococcus aureus* | 9.26 | *Staphylococcus aureus* | 2.24 |
| *Staphylococcus aureus* | 9.37 | *Staphylococcus aureus* | 2.03 |
| *Staphylococcus aureus* | 9.03 | *Staphylococcus aureus* | 2.07 |

| | | | |
|---|---|---|---|
| *Staphylococcus aureus* | 9.62 | *Staphylococcus aureus* | 2.29 |
| *Staphylococcus aureus* | 9.63 | *Staphylococcus aureus* | 2.33 |
| *Staphylococcus aureus* | 9.48 | *Staphylococcus aureus* | 2.22 |
| *Staphylococcus aureus* | 9.28 | *Staphylococcus aureus* | 2.28 |
| *Staphylococcus aureus* | 9.68 | *Staphylococcus aureus* | 2.46 |
| *Staphylococcus aureus* | 9.63 | *Staphylococcus aureus* | 2.41 |
| *Staphylococcus aureus* | 9.68 | *Staphylococcus aureus* | 2.44 |
| *Staphylococcus aureus* | 9.08 | *Staphylococcus aureus* | 2.11 |
| *Staphylococcus capitis* | 9.47 | *Staphylococcus capitis* | 2.14 |
| *Staphylococcus capitis* | 9.19 | *Staphylococcus capitis* | 2.05 |
| *Staphylococcus capitis* | 9.35 | *Staphylococcus capitis* | 2.12 |
| *Staphylococcus capitis* | 9.29 | *Staphylococcus capitis* | 2.11 |
| *Staphylococcus capitis* | 9.18 | *Staphylococcus capitis* | 2.04 |
| *Staphylococcus capitis* | 9.22 | *Staphylococcus capitis* | 2.12 |
| *Staphylococcus caprae* | 9.28 | *Staphylococcus caprae* | 2.03 |
| *Staphylococcus epidermidis* | 9.24 | *Staphylococcus epidermidis* | 2.09 |
| *Staphylococcus epidermidis* | 9.23 | *Staphylococcus epidermidis* | 2.0 |
| *Staphylococcus epidermidis* | 9.14 | *Staphylococcus epidermidis* | 2.13 |
| *Staphylococcus epidermidis* | 9.26 | *Staphylococcus epidermidis* | 2.07 |
| *Staphylococcus epidermidis* | 9.35 | *Staphylococcus epidermidis* | 2.03 |
| *Staphylococcus epidermidis* | 9.31 | *Staphylococcus epidermidis* | 2.16 |
| *Staphylococcus epidermidis* | 9.31 | *Staphylococcus epidermidis* | 2.22 |
| *Staphylococcus epidermidis* | 9.50 | *Staphylococcus epidermidis* | 2.16 |
| *Staphylococcus epidermidis* | 9.47 | *Staphylococcus epidermidis* | 2.06 |
| *Staphylococcus epidermidis* | 9.14 | *Staphylococcus epidermidis* | 2.1 |
| *Staphylococcus epidermidis* | 9.09 | *Staphylococcus epidermidis* | 2.0 |
| *Staphylococcus epidermidis* | 9.57 | *Staphylococcus epidermidis* | 2.12 |
| *Staphylococcus epidermidis* | 9.29 | *Staphylococcus epidermidis* | 2.02 |
| *Staphylococcus epidermidis* | 9.45 | *Staphylococcus epidermidis* | 2.07 |
| *Staphylococcus epidermidis* | 9.23 | *Staphylococcus epidermidis* | 2.02 |
| *Staphylococcus epidermidis* | 9.32 | *Staphylococcus epidermidis* | 2.16 |
| *Staphylococcus epidermidis* | 9.26 | *Staphylococcus epidermidis* | 2.01 |
| *Staphylococcus epidermidis* | 9.48 | *Staphylococcus epidermidis* | 2.02 |

| | | | |
|---|---|---|---|
| *Staphylococcus epidermidis* | 9.26 | *Staphylococcus epidermidis* | 2.1 |
| *Staphylococcus epidermidis* | 9.27 | *Staphylococcus epidermidis* | 2.02 |
| *Staphylococcus epidermidis* | 9.35 | *Staphylococcus epidermidis* | 2.24 |
| *Staphylococcus epidermidis* | 9.26 | *Staphylococcus epidermidis* | 2.02 |
| *Staphylococcus epidermidis* | 9.44 | *Staphylococcus epidermidis* | 2.08 |
| *Staphylococcus epidermidis* | 9.42 | *Staphylococcus epidermidis* | 2.03 |
| *Staphylococcus haemolyticus* | 9.05 | *Staphylococcus haemolyticus* | 2.06 |
| *Staphylococcus haemolyticus* | 9.61 | *Staphylococcus haemolyticus* | 2.08 |
| *Staphylococcus haemolyticus* | 9.53 | *Staphylococcus haemolyticus* | 2.09 |
| *Staphylococcus haemolyticus* | 9.36 | *Staphylococcus haemolyticus* | 2.05 |
| *Staphylococcus haemolyticus* | 9.06 | *Staphylococcus haemolyticus* | 2.04 |
| *Staphylococcus haemolyticus* | 9.59 | *Staphylococcus haemolyticus* | 2.10 |
| *Staphylococcus haemolyticus* | 9.00 | *Staphylococcus haemolyticus* | 2.05 |
| *Staphylococcus hominis* | 9.52 | *Staphylococcus hominis* | 2.12 |
| *Staphylococcus hominis* | 9.15 | *Staphylococcus hominis* | 2.01 |
| *Staphylococcus lugdunensis* | 9.27 | *Staphylococcus lugdunensis* | 2.09 |
| *Staphylococcus lugdunensis* | 9.58 | *Staphylococcus lugdunensis* | 2.2 |
| *Staphylococcus pasteuri* | 9.58 | *Staphylococcus pasteuri* | 2.19 |
| *Staphylococcus pseudintermedius* | 9.51 | *Staphylococcus pseudintermedius* | 2.02 |
| *Stenotrophomonas maltophilia* | 9.28 | *Stenotrophomonas maltophilia* | 2.44 |
| *Stenotrophomonas maltophilia* | 9.45 | *Stenotrophomonas maltophilia* | 2.07 |
| *Streptococcus agalactiae* | 9.60 | *Streptococcus agalactiae* | 2.3 |
| *Streptococcus agalactiae* | 9.59 | *Streptococcus agalactiae* | 2.18 |
| *Streptococcus agalactiae* | 9.6 | *Streptococcus agalactiae* | 2.31 |
| *Streptococcus agalactiae* | 9.64 | *Streptococcus agalactiae* | 2.22 |
| *Streptococcus agalactiae* | 9.73 | *Streptococcus agalactiae* | 2.31 |
| *Streptococcus agalactiae* | 9.73 | *Streptococcus agalactiae* | 2.4 |
| *Streptococcus agalactiae* | 9.6 | *Streptococcus agalactiae* | 2.15 |
| *Streptococcus agalactiae* | 9.51 | *Streptococcus agalactiae* | 2.21 |
| *Streptococcus agalactiae* | 9.47 | *Streptococcus agalactiae* | 2.16 |
| *Streptococcus anginosus* | 9.4 | *Streptococcus anginosus* | 2.13 |
| *Streptococcus anginosus* | 9.37 | *Streptococcus anginosus* | 2.11 |
| *Streptococcus anginosus* | 9.52 | *Streptococcus anginosus* | 2.23 |

| | | | | |
|---|---|---|---|---|
| *Streptococcus anginosus* | 9.52 | *Streptococcus anginosus* | 2.08 | |
| *Streptococcus anginosus* | 9.56 | *Streptococcus anginosus* | 2.01 | |
| *Streptococcus anginosus* | 9.51 | *Streptococcus anginosus* | 2.11 | |
| *Streptococcus anginosus* | 9.55 | *Streptococcus anginosus* | 2.06 | |
| *Streptococcus constellatus* | 9.55 | *Streptococcus constellatus* | 2.09 | |
| *Streptococcus dysgalactiae* | 9.12 | *Streptococcus dysgalactiae* | 2.23 | |
| *Streptococcus dysgalactiae* | 9.28 | *Streptococcus dysgalactiae* | 2.1 | |
| *Streptococcus gallolyticus* | 9.66 | *Streptococcus gallolyticus* | 2.25 | |
| *Streptococcus gallolyticus* | 9.46 | *Streptococcus gallolyticus* | 2.12 | |
| *Streptococcus gallolyticus* | 9.67 | *Streptococcus gallolyticus* | 2.21 | |
| *Streptococcus gallolyticus* | 9.72 | *Streptococcus gallolyticus* | 2.3 | |
| *Streptococcus gallolyticus* | 9.69 | *Streptococcus gallolyticus* | 2.26 | |
| *Streptococcus gallolyticus* | 9.05 | *Streptococcus gallolyticus* | 2.27 | |
| *Streptococcus gallolyticus* | 9.2 | *Streptococcus gallolyticus* | 2.22 | |
| *Streptococcus gordonii* | 9.27 | *Streptococcus gordonii* | 2.22 | |
| *Streptococcus infantis* | 9.36 | *Streptococcus infantis* | 1.78 | *Streptococcus infantis* |
| *Streptococcus intermedius* | 9.32 | *Streptococcus intermedius* | 2.36 | |
| *Streptococcus mitis* | 9.22 | *Streptococcus mitis_oralis* | 2.39 | *Streptococcus mitis* |
| *Streptococcus mitis* | 9.01 | *Streptococcus mitis_oralis* | 2.18 | *Streptococcus mitis* |
| *Streptococcus mitis* | 9.07 | *Streptococcus mitis_oralis* | 2.19 | *Streptococcus mitis* |
| *Streptococcus oralis* | 9.15 | *Streptococcus mitis_oralis* | 2.2 | *Streptococcus infantis* |
| *Streptococcus oralis* | 9.08 | *Streptococcus mitis_oralis* | 2.18 | *Streptococcus oralis* |
| *Streptococcus oralis* | 7.23 | *Streptococcus infantis* | 1.76 | *Streptococcus infantis* |
| *Streptococcus oralis* | 9.12 | *Streptococcus mitis_oralis* | 2.22 | *Streptococcus oralis* |
| *Streptococcus oralis* | 9.09 | *Streptococcus mitis_oralis* | 1.94 | *Streptococcus oralis* |
| *Streptococcus parasanguinis* | 9.03 | *Streptococcus parasanguinis* | 2.01 | |
| *Streptococcus pneumoniae* | 9.07 | *Streptococcus pneumoniae* | 2.19 | |
| *Streptococcus pneumoniae* | 9.34 | *Streptococcus pneumoniae* | 2.34 | |
| *Streptococcus pneumoniae* | 9.3 | *Streptococcus pneumoniae* | 2.39 | |
| *Streptococcus pseudopneumoniae* | 9.01 | *Streptococcus pseudopneumoniae* | 2.27 | |
| *Streptococcus pyogenes* | 9.14 | *Streptococcus dysgalactiae* | 2.17 | |
| *Streptococcus pyogenes* | 9.68 | *Streptococcus pyogenes* | 2.31 | |
| *Streptococcus pyogenes* | 9.28 | *Streptococcus pyogenes* | 2.12 | |

| | | | |
|---|---|---|---|
| *Streptococcus pyogenes* | 9.66 | *Streptococcus pyogenes* | 2.34 |
| *Streptococcus pyogenes* | 9.72 | *Streptococcus pyogenes* | 2.26 |
| *Streptococcus pyogenes* | 9.79 | *Streptococcus pyogenes* | 2.25 |
| *Streptococcus salivarius* | 9.63 | *Streptococcus salivarius* | 2.16 |
| *Veillonella atypica* | 9.33 | *Veillonella atypica* | 2.07 |
| *Yersinia enterocolitica* | 9.5 | *Yersinia enterocolitica* | 2.33 |
