# Supplementary material for: Evaluation of Autof MS2600 and MBT Smart MALDI-TOF MS Systems for Routine Identification of Clinical Bacteria and Yeasts
Source: Microorganisms. 2024 Feb 13;12(2):382. doi: 10.3390/microorganisms12020382 (PMC10892063; doi:10.3390/microorganisms12020382)
Supplement: Supplementary file 1 [file microorganisms-12-00382-s001.zip › Supplementary table S2. Identification score for yeast species.pdf]

MS2600 ID	SCORE Autobio	MBT smart	Score Bruker	Score UCSC Library	Sequence
Candida albicans	9.54	*Candida albicans*	no reliable	2.11	
Candida albicans	9.56	*Candida albicans*	no reliable	2.01	
Candida albicans	9.43	*Candida albicans*	no reliable	2.14	
Candida albicans	9.69	*Candida albicans*	1.96	2.0	
Candida albicans	9.46	*Candida albicans*	1.88	2.12	
Candida albicans	9.66	*Candida albicans*	no reliable	2.23	
Candida albicans	9.52	*Candida albicans*	2.15	2.2	
Candida albicans	9.64	*Candida albicans*	2.3	2.2	
Candida albicans	9.61	*Candida albicans*	2.08	2.01	
Candida albicans	9.67	*Candida albicans*	2.29	1.94	
Candida albicans	9.64	*Candida albicans*	2.09	1.86	
Candida albicans	9.34	*Candida albicans*	2.19	2.5	
Candida albicans	9.61	*Candida albicans*	2.21	2.12	
Candida albicans	9.14	*Candida albicans*	2.07	1.97	
Candida albicans	9.45	*Candida albicans*	2.12	2.07	
Candida albicans	9.09	*Candida albicans*	no reliable	2.12	
Candida albicans	9.3	*Candida albicans*	2.0	2.26	
Candida albicans	9.22	*Candida albicans*	1.78	2.3	
Candida albicans	9.55	*Candida albicans*	no reliable	2.38	
Candida albicans	9.65	*Candida albicans*	2.0	2.6	
Candida albicans	9.51	*Candida albicans*	2.11	1.96	
Candida albicans	9.15	*Candida albicans*	2.03	2.14	
Candida albicans	9.7	*Candida albicans*	2.06	2.24	
Candida albicans	9.6	*Candida albicans*	1.70	2.25	
Candida albicans	9.7	*Candida albicans*	no reliable	2.09	
Candida albicans	9.52	*Candida albicans*	no reliable	2.09	
Candida albicans	9.72	*Candida albicans*	2.15	2.17	
Candida albicans	9.72	*Candida albicans*	no reliable	2.14	
Candida albicans	9.71	*Candida albicans*	1.87	2.11	
Candida albicans	9.46	*Candida albicans*	2.21	2.36	
Candida albicans	9.62	*Candida albicans*	1.91	2.17	
Candida albicans	9.52	*Candida albicans*	2.04	2.21	
Candida albicans	9.56	*Candida albicans*	2.07	2.13	
Candida albicans	9.16	*Candida albicans*	no reliable	2.21	
Candida albicans	9.57	*Candida albicans*	2.14	2.31	
Candida auris	9.33	*Candida auris*	no reliable	2.05	
Candida auris	9.07	*Candida auris*	no reliable	2.05	
Candida auris	9.47	*Candida auris*	no reliable	2.01	
Candida auris	9.19	*Candida auris*	no reliable	2.06	
Candida dubliniensis	9.08	*Candida dubliniensis*	2.13	1.95	
Candida dubliniensis	9.09	*Candida dubliniensis*	2.28	2.02	
Candida glabrata	9.21	*Candida glabrata*	2.25	2.45	
Candida glabrata	9.21	*Candida glabrata*	2.23	2.38	
Candida glabrata	9.15	*Candida glabrata*	2.11	2.04	

Candida glabrata	9.25	*Candida glabrata*	2.26	2.36	
Candida glabrata	9.01	*Candida glabrata*	2.45	2.52	
Candida glabrata	9.03	*Candida glabrata*	2.04	2.13	
Candida glabrata	9.21	*Candida glabrata*	2.08	1.98	
Candida glabrata	9.21	*Candida glabrata*	2.01	2.02	
Candida glabrata	9.08	*Candida glabrata*	2.18	2.04	
Candida glabrata	9.33	*Candida glabrata*	2.2	2.27	
Candida haemulonii	9.22	*Candida haemulonii*	no reliable	no reliable	*Candida haemulonii*
Candida haemulonii	9.1	*Candida haemulonii*	no reliable	no reliable	*Candida haemulonii*
Candida kefyr(Kluyveromyces marxianus)	9.14	*Candida kefyr*	1.80	2.01	
Candida krusei(Issatchenkia orientalis)	9.05	*Candida krusei(Issatchenkia orientalis)*	no reliable	2.04	
Candida krusei(Issatchenkia orientalis)	9.04	*Candida krusei(Issatchenkia orientalis)*	no reliable	2.01	
Candida krusei(Issatchenkia orientalis)	9.06	*Candida krusei(Issatchenkia orientalis)*	no reliable	2.08	
Candida krusei(Issatchenkia orientalis)	9.17	*Candida krusei(Issatchenkia orientalis)*	1.88	2.11	
Candida krusei(Issatchenkia orientalis)	9.08	*Candida krusei(Issatchenkia orientalis)*	no reliable	2.01	
Candida metapsilosis	9.23	*Candida metapsilosis*	no reliable	2.0	
Candida metapsilosis	9.23	*Candida metapsilosis*	no reliable	2.29	
Candida metapsilosis	9.01	*Candida metapsilosis*	no reliable	2.24	
Candida metapsilosis	9.04	*Candida metapsilosis*	1.97	2.25	
Candida metapsilosis	9.14	*Candida metapsilosis*	no reliable	2.19	
Candida metapsilosis	9.04	*Candida metapsilosis*	no reliable	2.30	
Candida nivariensis	9.16	*Candida nivariensis*	no reliable	2.06	
Candida nivariensis	9.34	*Candida nivariensis*	no reliable	2.02	
Candida norvegensis (Pichia norvegensis)	9.33	*Candida norvegensis*	1.96	2.08	
Candida norvegensis (Pichia norvegensis)	9.12	*Candida norvegensis*	2.11	1.82	
Candida orthopsilosis	9.25	*Candida orthopsilosis*	no reliable	2.22	
Candida ortopsilosis	9.22	*Candida orthopsilosis*	no reliable	2.04	
Candida ortopsilosis	9.24	*Candida orthopsilosis*	no reliable	2.02	
Candida ortopsilosis	9.18	*Candida orthopsilosis*	no reliable	2.22	
Candida ortopsilosis	9.05	*Candida orthopsilosis*	no reliable	2.26	
Candida parapsilosis	9.18	*Candida parapsilosis*	1.85	2.08	
Candida parapsilosis	9.04	*Candida parapsilosis*	1.75	2.11	
Candida parapsilosis	9.49	*Candida parapsilosis*	2.01	2.3	
Candida parapsilosis	9.04	*Candida parapsilosis*	1.78	2.04	
Candida tropicalis	9.1	*Candida tropicalis*	2.1	1.93	
Candida tropicalis	9.45	*Candida tropicalis*	2.02	1.92	
Candida tropicalis	9.32	*Candida tropicalis*	2.0	2.3	
Candida tropicalis	9.15	*Candida tropicalis*	2.22	1.70	
Candida tropicalis	9.5	*Candida tropicalis*	2.12	2.04	
Candida tropicalis	9.37	*Candida tropicalis*	no reliable	2.02	
Candida tropicalis	9.32	*Candida tropicalis*	2.01	1.91	
Cryptococcus neoformans	9.06	*Cryptococcus neoformans*	1.86	2.05	
Cryptococcus neoformans	9.02	*Cryptococcus neoformans*	1.75	2.16	
Cryptococcus neoformans	9.10	*Cryptococcus neoformans*	1.86	2.23	
Cryptococcus neoformans	9.12	*Cryptococcus neoformans*	1.9	2.05	

Cryptococcus neoformans	7.89	no reliable	no reliable	-	*Cryptococcus neoformans*
Rhodotorula mucillaginosa	9.21	*Rhodotorula mucillaginosa*	1.86	2.06	*Rhodotorula mucillaginosa*
Rhodotorula mucillaginosa	9.12	*Rhodotorula mucillaginosa*	1.97	2.23	*Rhodotorula mucillaginosa*
Rhodotorula mucillaginosa	9.57	*Rhodotorula mucillaginosa*	1.94	2.19	
Rhodotorula mucillaginosa	9.3	*Rhodotorula mucillaginosa*	1.71	2.17	
Rhodotorula mucillaginosa	9.37	*Rhodotorula mucillaginosa*	no reliable	2.14	
Saccharomyces cerevisiae	9.02	*Candida robusta*	no reliable	2.05	
Saccharomyces cerevisiae	9.08	*Saccharomyces cerevisiae*	no reliable	2.01	
Saccharomyces cerevisiae	9.09	*Candida robusta*	no reliable	2.04	
Saprochaete clavata	no reliable	*Saprochaete clavata*	no reliable	no reliable	*Saprochaete clavata*
Trichosporon asahii	9.30	*Trichosporon asahii*	1.79	2.09	
Trichosporon asahii	9.18	*Trichosporon asahii*	1.87	2.14	
